# Model-Based Computational Analysis on the Effectiveness of Enhanced Recovery after Surgery in the Operating Room with Nursing

**DOI:** 10.3389/fsurg.2022.922684

**Published:** 2022-05-18

**Authors:** Wenji Li, Shu Huang, Yong Xie, Guanyu Chen, Jun Yuan, Yun Yang

**Affiliations:** ^1^ The First Affiliated Hospital, Sun Yat-sen University, Guangzhou, China; ^2^ Department of Orthopedics, Hunan Provincial People’s Hospital (The First-Affiliated Hospital of Hunan Normal University), Changsha, China

**Keywords:** surgery, operating room nursing, enhanced recovery after surgery, complication, satisfaction

## Abstract

**Objective:**

In order to better understand the relative surgical process, this work used a model-based computational analysis on the effectiveness of enhanced recovery after surgery (ERAS) in the operating room with nursing.

**Methods:**

A total of 360 surgical patients in the First Affiliated Hospital, Sun Yat-sen University, from the period June 2020 to March 2021, were randomly divided into two groups, namely, observation group and control group, with 180 cases in each group. Routine nursing was used in the control group, while ERAS was implemented in the observation group from the point of view of four aspects, namely, preoperative visit, intraoperative cooperation, postoperative return visit, and psychological intervention.

**Results:**

Postoperative complications, average hospital stay, nursing satisfaction, and postoperative quality of life in the observation group were significantly better than those in the control group (all *p *< 0.05).

**Conclusion:**

The application of ERAS for surgical patients can enhance team awareness, optimize the process of cooperation, reduce surgical complications and improve nursing quality, and prognosis, and it is worth popularizing in the operating room.

## Introduction

Surgery refers to the treatment that doctors use with knives, scissors, needles, and other medical instruments to cut off and sew parts of the human body to maintain or even save the patient’s health. This surgical treatment is commonly known as “operation”. The purpose is to treat or diagnose diseases to improve the body’s function and shape, such as removing diseased tissues (1, 2), repairing injuries (3, 4), and organ transplantation (5, 6). Early surgery is limited to cutting and suturing on the body surface by simple manual methods such as abscess drainage, tumor resection, and trauma suturing. With the development of surgery, the field of surgery has been expanding, and today, it can be performed in any part of the human body (7–10). In addition, it has been reported that surgery has greater efficacy than non-surgical treatments in curing some human diseases (11, 12).

**Figure 1:**
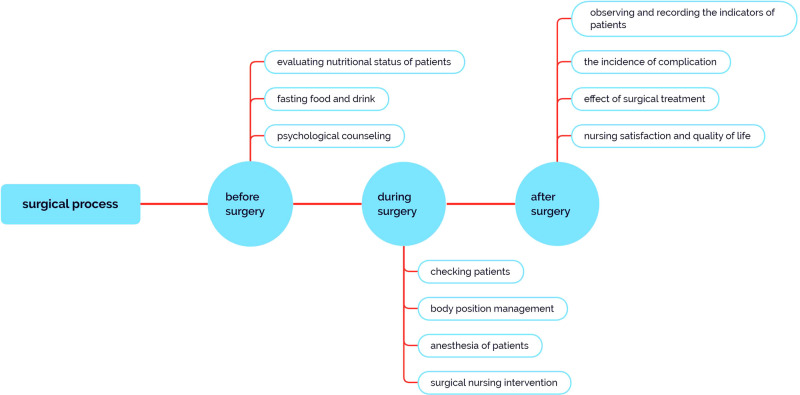
General surgical process during the perioperative period.

However, various intraoperative complications and postoperative complications may occur due to injury, bleeding, or infection caused by surgical treatment (13–15). In addition, when patients undergo surgery, they have to experience the stimulation of anesthesia and surgical trauma. Their body will be in a state of stress, which will lead to both psychological and physiological burden (16). Therefore, some kind of good and effective perioperative nursing is required to provide patients with holistic physical and mental care so that they can successfully spend their perioperative period in the best frame of mind (Figure 1). Such nursing also plays an extremely important role in preventing or reducing postoperative complications (17).

The theory of Enhanced Recovery after Surgery (ERAS) was proposed systematically by Danish surgeon Professor Kehlet (18) for the first time in 1997, which refers to the adoption of a series of perioperative optimization measures with evidence-based medical evidence to block or reduce the stress response of the body. It can promote the accelerated recovery of patients after surgery and achieve the purpose of shortening the patient’s hospitalization time so as to reduce postoperative complications and also the risk of readmission and death (19). It has been verified that ERAS has a very positive application (20, 21). The purpose of this study is to research and analyze the effect of ERAS on perioperative nursing and provide a reference for further study.

## Materials and Methods

### General Description

A total of 360 (223 males and 137 females) surgical patients in the First Affiliated Hospital, Sun Yat-sen University, from the period June 2020 to March 2021, were selected as the research objects. All the selected patients underwent elective surgery, following which all of them could actively cooperate with perioperative nursing guidance. The whole study was carried out with the informed consent of these patients and approved by the hospital ethics committee.

All patients were randomly divided into two groups, 180 in each group. Of these, 118 males and 62 females with age ranging from 61 to 78 years and an average of (62.50 ± 15.60) years were in the observation group, in which ERAS was implemented in the form of preoperative visit, intraoperative cooperation, postoperative return visit, and psychological intervention. A total of 105 males and 75 females with age ranging from 51 to 81 years and an average of (62.70 ± 14.60) years were in the control group, in which routine nursing was implemented. There was no significant difference between the two groups in the general data such as gender, age, and gastrointestinal diseases (all *p *< 0.05), which indicated that they were comparable in this study.

### Materials

#### Routine Nursing

The control group was given routine nursing care. Preoperative nursing was carried out for the purpose of education. Patients were required to fast for 8–12 h and abstain from drinking for 4  h (7). After entering the operating room, the patients were checked, and venous access was established. After general anesthesia, the patients were placed in the operating position. They could eat after anal exhaust, the complications of which were observed and recorded.

#### ERAS Pathway

The observation group received routine nursing and the corresponding nursing intervention combined with ERAS, including preoperative nursing, operation room nursing, and postoperative nursing, which are described in the following paragraphs.

##### Preoperative Nursing

In the ERAS pathway, good preoperative preparation and psychological nursing play a key role in the smooth conduct of operation. Nurses should visit patients 1 day before operation and give them appropriate diet and psychological nursing.

###### 2.2.2.1.1 Psychological Nursing

Surgery is an invasive operation, which causes serious psychological burden to patients. Anxiety is a common psychological condition of patients before surgery. Psychological counseling should be done well before surgery to enhance the confidence of patients during surgery.

###### 2.2.2.1.2 Self-Care Ability

The self-care ability of the patients were evaluated according to the inputs provided by the patients in the self-care ability evaluation form. Self-care ability was divided into four levels, namely, no dependence, mild dependence, moderate dependence, and severe dependence. The self-care ability of these levels was evaluated as none needed for care, a few needed care, most needed care, and all needed care, respectively. Dynamic evaluation was made according to the changes in the patients’ condition and nursing levels, and corresponding nursing measures such as secondary care, primary care, and special care were implemented.

###### 2.2.2.1.3 Diet Nursing

The nutritional status of the patients was evaluated. Patients without gastrointestinal motility disorder were required to fast solid food for 6  h and liquid food for 2  h before operation. They were required to take two bottles of “Suqian beverage” (a kind of maltose fructose drink made in China) of approximately 800  ml orally at 22:00  and one bottle of approximately 400  ml 2  h before operation. Reducing the hunger, thirst and anxiety of patients can lower the incidence of postoperative nausea and vomiting, which will accelerate their recovery.

##### Operating Room Nursing

The bladder of the patients should be confirmed empty while the nurse brings them into the operating room. An equilibrium liquid of approximately 30 drops/min was given to the patients after confirming the standby state of the indwelling needle and slowly dripping it for maintenance (22, 23). The roving nurse and the workers jointly verified the general information of the patients and handed over their intraoperative medication, imaging data, special supplies, and medical records. After signing the printed operation handover form, the patients were sent to the operating room.

The patients were under anesthesia during the operation. Excessive blood loss and fluid loss may be caused by long operation time and trauma. Therefore, it is highly important to implement operating room nursing intervention in the ERAS pathway. The infusion channel should be reasonable, and the appropriate venous catheter should be selected. In case of significant blood loss and fluid loss during the operation, the large-diameter venous channel and central venous catheters anti-infection catheter should be selected and the three-way pipe should be managed well. It is reported that the pollution rate of the three-way pipe during the operation can reach 23%. The integrated board was used to prevent infection. In addition, body position management should be standardized. The exposed field should be convenient for the operator to conduct the operation. The body should be placed gently and the functional position should be maintained after the body is placed. Personalized body position should be adopted to avoid skin damage and nerve damage. Physical preventive measures such as elastic socks and intermittent pressurizing devices can be used to avoid low blood volume. A specialist group should be set up, with a specialist nurse as the team leader. Daily staff should be arranged by the specialist group every day. Operational materials should be prepared well according to the doctor’s instructions, and the staff should actively cooperate with the surgeons to shorten the operation time.

##### Postoperative Nursing

The patients went back to the ward after anesthesia. Evaluation and handover were made according to the observation record sheet of the anesthesia recovery room (PACU). The handover contents mainly include the following: identity confirmation, vital signs, consciousness, respiration, circulation, oxygen saturation, the patient’s limb mobility, oral and lip color, infusion, urinary catheter, medication, drainage and wound dressing, and skin.

### Observation Indicators

The incidence of postoperative complications, treatment effect, nursing satisfaction, and quality of life were compared between the two groups (22–24). According to the questionnaire of patient satisfaction in the operating room developed by our hospital, the patients scored on the spot to judge their nursing satisfaction during the postoperative return visit. Satisfaction rate = very satisfied + satisfied (the total number of people).

### Statistical Method

SPSS 26.0 statistical software was used to analyze the data. The measurement data were expressed by average $\left(\bar{x}\pm s\right)$ and the *t*-test was used. The counting data were expressed by percentage (%) and the *X^2^* test was used. The difference was statistically significant (*p *< 0.05).

## Results and Discussions

As shown in Table 1, complications such as skin injury, shiver, and incision infection occurred in both groups, which include 13 cases in the observation group (7.22%) and 35 cases in the control group (19.44%). The number of patients with complications in the observation group was significantly less than those in the control group, which indicated better nursing effect on ERAS (*p *< 0.05). One of the concepts of ERAS is to reduce the incidence of postoperative complications and promote the recovery of patients’ physical and psychological health (25), which is consistent with the results in Table 1. Nursing staff made a comprehensive evaluation of the preoperative visits of the patients in the observation group before the operation. The infusion pipeline was well managed during the operation, and the operation position was correctly placed to prevent hypothermia. In addition, a series of nursing interventions to prevent deep vein thrombosis and control incision infection were adopted, which significantly reduced the complication rate of the patients.

**Table 1:** Comparison of postoperative complications between two groups (*n* = 360, %).

Groups	Skin injury	Shiver	Incision infection	Incidence rate (%)
Observation group	4	6	3	7.22
Control group	12	14	9	19.44
*X*^2^-value	8.226	8.126	4.232	
*p*-Value	0.005*	0.002*	0.001*	

* *p < 0.01.*

Generally, surgical patients experienced moderate and severe pain. Good postoperative analgesia can relieve their tension and anxiety. In the ERAS pathway, a return visit was made to correctly evaluate the patients’ pain after the operation. It is beneficial for wound healing and will speed up recovery if analgesia is given in a preventive, timely, and multimode manner (26). ERAS has been shown to allow patients to move out of bed sooner (27, 28) and reduce the length of stay in hospital (29, 30). From Table 2, it can be seen that the patients in the observation group were significantly better than those in the control group in terms of exhaust time, free movement time out of bed, and average length of hospital stay (*p *< 0.05), which showed consistency with the previous report.

**Table 2:** Comparison of therapeutic effects between two groups (*n* = 360, $\bar{x}\pm s$).

Groups	Anal exhaust time (h)	free movement time out of bed (h)	Average length of hospital stay (days)
Observation group	33.30 ± 3.26*	19.80 ± 4.26*	9.07 ± 1.26*
Control group	64.50 ± 3.28*	33.72 ± 2.32*	18.01 ± 2.26

*p < 0.05.

As shown in Table 3, patients’ satisfaction with nursing in the observation group (98.30%) was significantly higher than that in the control group (85.00%), and the difference was statistically significant (*p *< 0.05). Compared with patients who underwent routine nursing, the time of fasting food and drink of those who adopted ERAS was shortened. The patients’ hunger, panic, and fear caused by long-term fasting were avoided. Effective communication with the patients was made before the operation. The patients could more clearly understand the purpose and time of fasting so that they could more actively cooperate during the perioperative period. Therefore, nursing satisfaction was improved (31).

**Table 3:** Comparison of nursing satisfaction between two groups (*n* = 360, %).

Groups	Number	Very satisfied	Satisfied	Dissatisfied	Degree of satisfaction (%)
Observation group	180	108	69	3	98.30*
Control group	180	58	95	27	85.00*

* *p < 0.05.*

Quality of life was positively correlated with the score. The higher the total scores, the higher the quality of life. As shown in Table 4, the scores of quality of life after nursing in the observation group were significantly higher than those in the control group (*p *< 0.05) after psychological intervention. It was reported that ERAS can significantly improve patients’ mental health and physical health, which was basically consistent with the conclusion in Table 4 of this study (32). Moreover, psychological intervention can improve the patient compliance following the ERAS after operation.

**Table 4:** Comparison of quality of life after nursing care between two groups (*n* = 360, $\bar{x}\pm s$).

Groups	Physiological functioning	General health	Mental health	Vitality	Bodily pain	Role-emotional	Social functioning
Observation group	87.35 ± 6.31	78.39 ± 5.76	70.20 ± 5.12	82.14 ± 6.21	75.21 ± 5.21	79.02 ± 5.21	81.62 ± 6.06
Control group	72.37 ± 6.35	72.32 ± 5.26	63.21 ± 4.98	74.21 ± 6.09	70.11 ± 5.31	73.21 ± 5.06	76.22 ± 5.26
*t*-Value	9.903	5.721	4.226	4.781	4.919	5.121	5.266
*p*-Value	<0.001*	<0.001*	<0.001*	<0.001*	<0.001*	<0.001*	<0.001*

* *p < 0.01.*

## Conclusions

In this study, the effects of routine nursing and ERAS on perioperative nursing were compared. The results indicated that the ERAS pathway can not only reduce postoperative complications and shorten the length of hospital stay, but also improve patients’ quality of life. From this study, we can see that for patients, the application of the ERAS theory during the perioperative period can shorten the operation time and reduce their postoperative complications so as to improve the prognosis and enhance their overall satisfaction with the quality of care. For surgeons, ERAS can enhance the awareness of the operation team and optimize the operation process of cooperation, which is worth popularizing.

With the development of medical technology, minimally invasive surgery and precise medications have led to fewer contraindications for surgical treatment. Surgery, as the main method of invasive treatment, has a great impact on the status of patients’ psychology and physiology. In order to alleviate patients’ anxiety and fear before operation, improve nursing quality, and reduce postoperative complications, operating room nursing staff are required to keep pace with the times and garner new ideas to serve patients. However, due to a wide range of departments involved in ERAS, multiteam and multidisciplinary assistance are required. In our study, ERAS proved to be an effective way to help patients recover quickly and comprehensively, thus providing a good reference and theoretical basis for studying ERAS and changing traditional nursing concepts to devise more effective nursing measures.

## Data Availability

The original contributions presented in the study are included in the article/supplementary material; further inquiries can be directed to the corresponding author/s.
